# Neurocognitive Challenges During Drop Vertical Jumps Increase Sensitivity to Differentiate Atypical Landing Mechanics and Jump Height in Individuals With Anterior Cruciate Ligament Reconstruction

**DOI:** 10.1177/03635465251346145

**Published:** 2025-06-07

**Authors:** Andrew Strong, Jonas L. Markström

**Affiliations:** * Department of Community Medicine and Rehabilitation, Unit of Physiotherapy, Umeå University, Umeå, Sweden; Investigation conducted at the Department of Community Medicine and Rehabilitation, Unit of Physiotherapy, Umeå University, Umeå, Sweden

**Keywords:** knee, ligaments, ACL, physical therapy/rehabilitation, general sports trauma, injury prevention

## Abstract

**Background:**

Athletes with anterior cruciate ligament reconstruction (ACLR) have high rates of secondary injury. Insufficient return-to-sport screening may be due to standard functional tests not resembling chaotic sporting environments where injuries occur. Neurocognitive deficits among individuals with ACLR indicate that cognitive challenges during screening tests may better reveal atypical movement mechanics.

**Hypothesis:**

Adding secondary cognitive tasks to drop vertical jumps (DVJs) would increase between-group differences in landing mechanics and jump height compared with the standard DVJ.

**Study Design:**

Controlled laboratory study.

**Methods:**

Forty sports-active individuals 24.9 ± 16.1 months after unilateral ACLR and 40 uninjured controls (both groups 50% female) performed DVJs; downward- or upward-pointing arrows indicated whether to drop only or complete the vertical jump. Conditions were (1) black arrow presented before drop and (2) black or red arrow presented during drop (red arrow pointing in opposite direction of requested motor action) together with a memory task involving letter recalling. Jump height and biomechanical time-series data from an 8-camera motion capture system and 2 force plates during the first 100 ms of landing were compared between groups using conventional and functional *t* tests, respectively.

**Results:**

For the standard DVJ, the ACLR group had significantly less hip power and more hip abduction moment for the injured leg and uninjured leg, respectively, compared with controls. For the DVJ with secondary cognitive tasks, the ACLR group again showed significantly less hip power and more hip abduction moment but also less knee power, knee flexion moment, ankle power, and ankle dorsiflexion moment and lower jump height than controls.

**Conclusion:**

The addition of secondary cognitive tasks during DVJs elicited further significant differences in landing mechanics and jump performance among athletes with ACLR compared with uninjured athletes than were found for the standard DVJ. The aberrant biomechanical outcomes for the ACLR group indicate an incomplete rehabilitation.

**Clinical Relevance:**

The greater between-group differences in landing mechanics and jump height when adding secondary cognitive tasks to a DVJ indicate a need to provide neurocognitive challenges in rehabilitation and return-to-sport screening as a first step toward improved rehabilitation outcomes and more ecologically valid testing.

Anterior cruciate ligament (ACL) rupture is the most common injury leading to the cessation of sports participation among high school athletes in the United States.^35^ Treatment strategies and criteria-based screening protocols are continually being developed to ensure readiness for return to sport (RTS).^22^ However, many do not return to their previous level of sport,^3,12^ and of those who do, the secondary ACL injury rate has been reported at an alarming 23%.^36^

Most ACL injuries occur in noncontact situations when landing from a jump, stopping suddenly from a high-velocity run, or changing direction. The abrupt increase in ground-reaction forces and aberrant landing mechanics excessively strain the ACL until it ruptures. Injuries often transpire in chaotic sporting environments where athletes must process rapidly changing information, such as opponent behavior and ball trajectory, to guide fast decision-making. Efficient neurocognitive processing is therefore crucial for appropriate motor behavior during sport.

Screening for RTS after ACL injury commonly includes the assessment of isolated functional tests such as preplanned jumps and hops. In addition to discrete performance measures (eg, height and distance), assessment of biomechanical outcomes from landings is recommended.^21^ The high secondary ACL injury rates indicate, however, that not all individuals with heightened risk for injury are identified. It has therefore been recommended to add neurocognitive tasks to planned and unplanned functional tests as part of RTS testing to better resemble the demands of sport.^7,15^ In support of this, a recent systematic review^38^ among uninjured athletes found acute decreases in performance when cognitive and motor tasks were performed concurrently compared with when performed in isolation, an effect called cognitive-motor interference (CMi).^24^

Evidence of central nervous system adaptations after ACL injury^10^ has accumulated over recent years. These adaptations are believed to be due, in part, to the loss of sensory receptors found in the intact ligament.^1^ Deficits in neurocognitive functioning have also been found among male football (soccer) players 6 to 12 months after unilateral ACL reconstruction (ACLR) compared with matched controls, regardless of whether traditional RTS criteria were achieved.^19^ In partial support of these combined findings, a systematic review found limited evidence of greater CMi for individuals with ACL injury compared with uninjured controls for some dual tasks combining postural control or gait with cognitive challenges.^28^ However, the difficulty level of the task elements was indicated as being influential on the outcomes, and the inclusion of more athletic tasks, such as jumps, was noted as an important gap in the literature.

Recent evidence of CMi for individuals with ACLR during athletic tasks has begun to emerge. For unplanned landings among football (soccer) players, adding cognitive components was observed in 1 study not to influence landing mechanics^2^ but did so in another.^13^ For single-leg hop for distance landings, a working memory task has been shown to influence coordination variability in both legs of uninjured controls and the uninjured leg of individuals with ACLR but not the leg with ACLR, suggesting enhanced cognitive control due to the injury.^14^ Among athletes who had returned to sport after ACLR, we recently showed that adding secondary cognitive tasks to a drop vertical jump (DVJ) led to altered landing mechanics that have been associated with an increased ACL injury risk and thus indicate inadequate rehabilitation.^32^ Taken together, evidence indicates that current RTS testing may fail to identify critical neurocognitive consequences of ACL injury, but further evidence is required to investigate the role of cognitive-motor dual-task tests to identify atypical landing mechanics after ACL injury.

The aim of this study was to investigate differences in landing mechanics and jump height between individuals with ACLR compared with uninjured controls for DVJs with and without secondary cognitive tasks. Based on evidence suggesting that individuals with ACL injury may experience greater challenges for cognitive-motor dual tasks compared with controls (ie, increased CMi), we hypothesized that adding secondary cognitive tasks to DVJs would increase between-group differences in landing mechanics and jump height compared with the standard DVJ.

## Methods

### Study Design

This controlled laboratory study was approved by the National Ethical Review Authority (Dnr. 2023-00342-01). The study was conducted in line with the ethical principles stated in the Declaration of Helsinki, and participants gave prior written informed consent to participate.

### Participants

We recruited 40 individuals with ACLR (50% males; ACLR group) and 40 uninjured individuals (50% males; control group) (Table 1). Participants were recruited between April 2023 and April 2024 from the orthopaedic clinic of the regional hospital through a register as well as via advertisements, local contacts, and word-of-mouth. The inclusion criteria were age 15 to 36 years (relevant for higher level competition), a Tegner activity scale^34^ rating of ≥6 out of 10, a return to sports that involve unpredictable rapid directional changes (eg, team sports and ball sports), confidence in performing maximal hop and strength tests, a unilateral ACL injury with an ipsilateral hamstring graft (standard national practice), maximum 5 years from ACLR, no concomitant injuries (eg, complete tear of another knee ligament, significant meniscal or articular damage), no severe ankle sprain within the past 6 months, and no other conditions that could impair jumping ability. The same criteria were applied to control participants recruited through advertisements, local contacts, and word-of-mouth.

**Table 1:** Participant Demographic Characteristics*^*a*^*

Variable	ACLR	Controls
Male/female, n	20/20	20/20
Age, y	25.7 ± 5.5	22.0 ± 4.9
Body mass index, kg/m^2^	24.5 ± 2.7	23.5 ± 3.3
Time from ACLR to test, mo	24.9 ± 16.1	—

a Data are presented as mean ± SD unless otherwise noted. ACLR, anterior cruciate ligament reconstruction.

### Sample Size Calculation

A power analysis based on means and standard deviations of knee moment and power from a pilot study (our own, unpublished data) for the same DVJ dual-task paradigm including 6 athletes with ACLR and 6 uninjured athletes indicated that 15 to 29 individuals per group were required to detect between-group differences (independent *t* test; 2-sided hypothesis; power = 90%; alpha = 5%). We recruited 40 individuals per group to attain power for time series analyses (the 12 participants in the pilot study were not included in this study).

### Jump Testing

Participants wore their own sports attire, including shoes and short tights; women wore a sports bra. They began with a standardized warm-up consisting of 2 circuits of 6 squats, 3 lunges per leg, and 3 squat jumps. Three or 4 practice trials of the DVJ were also performed before each DVJ test. The same test leader (J.L.M.) provided instructions to all participants. Participants then performed the DVJs both without (DVJ) and with (DVJcog) secondary cognitive tasks designed to challenge attention, short-term memory, fast decision making, and inhibitory control. These cognitive elements reflect the fundamental demands of both recreational and elite sports, requiring athletes to stay attentive, quickly adapt to their surroundings, and process information for future decision making. The order of these DVJ tests was randomized. Participants were given the following instructions for both DVJs, which emphasized an external focus of attention: “Land softly and as quietly as possible, and if jumping after the landing, push the ground away as hard as you can to jump as high as possible.”

#### DVJ

Participants stood on a 35 cm–high wooden box and saw a black arrow pointing up or down on a television screen (~5 m away). They then dropped down at a time of their choosing (~50 cm forward) and performed the correct motor action, where a black arrow pointing up indicated to land and immediately jump as high as possible and a black arrow pointing down indicated to land only (ie, planned motor action). Participants completed 3 trials for each arrow in a randomized order, with a counterbalanced total such that upward- and downward-pointing black arrows were displayed 3 times each (n = 6).

#### DVJcog

Participants stood on the box and first had 5 seconds to memorize the position of the letters A to F on the television screen. Participants then dropped down at a time of their choosing (~50 cm forward), and as soon as their heel lifted from the box, 1 arrow was shown with 1 letter inside for 1 second (element of fast decision-making due to the unplanned motor action). In addition to the black arrows, an upward- or downward-pointing red arrow indicated to perform the opposite action compared with the black arrows (element of inhibitory control). Participants were to memorize the letter, perform the correct motor action, and then recall where the letter in the arrow was positioned (elements of attention and short-term memory). The order of the letters A through F and the letter inside the arrow were randomized between trials. Participants completed 3 trials for each arrow in a randomized order, with a counterbalanced total such that upward- and downward-pointing black and red arrows were displayed 3 times each (n = 12 trials). If a participant performed an incorrect motor action during the DVJcog, the trial was repeated (without the participant’s knowledge) after the 12 trials to ensure 3 successful trials per arrow, allowing for the calculation of individual mean time series under equal conditions.

### Instruments and Data Processing

Participants landed with 1 foot on each of 2 force plates (1200 Hz; Model 9260AA, Kistler Instrument AG) placed side by side at ground level and concealed by modular walkway elements of the same color. The force data were synchronized with an 8-camera motion capture system (240 Hz; Oqus 300, Qualisys AB), which tracked the movements of passive reflective markers attached on the sacrum (midpoint of the right and left posterior superior iliac spine) and bilaterally on the shoulders (superior surface of the midpoint of the acromion process), crista (most superior and lateral point of the crests), anterior superior iliac spines, trochanter majors (most prominent aspect), lateral and medial epicondyles, tuberositas tibiae (most prominent aspect), fibular heads (most lateral and prominent aspect), medial malleoli (most medial and prominent aspect), lateral malleoli (most lateral and prominent aspect), calcanei (posterior aspect, both at proximal and distal ends of the midline), the proximal base of the first metatarsals, medial aspect of the first metatarsals, and fifth metatarsal heads. Participants also wore rigid clusters with 4 markers on each thigh, with the 2 medial markers positioned in the middle of the thigh below the patellar midpoint while standing, to minimize the effect of soft tissue artifacts.^9^ After a standing calibration file was recorded, markers on the sacrum, trochanter majors, lateral and medial epicondyles, and medial malleoli were removed.

The markers were tracked using Qualisys Track Manager software (Version 2019.3) and exported to Visual 3D software (Version 5.02.30; C-Motion), where their movements were filtered at 15 Hz using a critically damped digital filter. These data were used to create an 8-segment, 6 degrees of freedom model consisting of the trunk, pelvis, thighs, shanks, and feet. Joint kinematics were calculated using the Cardan rotation sequence XYZ (X, mediolateral axis; Y, anteroposterior axis; Z, longitudinal axis),^8^ based on the movement of the distal segment relative to the proximal segment. Hip joint centers were defined using a functional joint method based on hip circumduction movements, with thigh cluster markers and the pelvis as a reference.^30^ Knee and ankle joint centers were defined as the midpoint between the markers placed on the femoral epicondyles and malleoli, respectively. Joint kinetics were calculated through inverse dynamics using the resultant moments approach,^4^ with coordinate systems resolved in the proximal segment’s coordinate system. Moments were normalized to body mass and expressed as external moments. Segment masses were calculated based on Dempster’s proportions.^11^ Both kinematic and kinetic data were filtered at 15 Hz using a fourth-order, bidirectional, zero-lag, low-pass Butterworth digital filter before being used to calculate the outcome variables.

The arrows and letters displayed during the DVJs were triggered by a custom-made system that detected events using a laser-ranging time-of-flight sensor (ST VL53L1X) positioned on the box directed toward participants’ heels (thus activated when lifted) and a microcontroller (Arduino UNO). The hardware communicated these events through a serial interface to software on a computer. The mean time (with standard deviation) between the appearance of the arrows on the television and when participants landed was 280 ± 64 ms. This time was calculated using an optical sensor that recorded when the arrow appeared and when the vertical ground-reaction force (vGRF) first exceeded 20 N on either force plate. This time varied across trials because the jump down from the box varied. The microcontroller firmware was written in Arduino language and the computer software in C# language.

### Outcome Variables

Time series data were analyzed during the initial 100 ms of the first landing, focusing on sagittal and frontal plane hip and knee angles and moments; sagittal plane ankle angle and moment; and hip, knee, and ankle powers. These outcomes were chosen because they are relevant to the DVJ’s forward movement and provide information on incomplete rehabilitation.^21^ This landing was identified when the force plate data on either force plate exceeded 20 N. The data were discretized into 101 points, and individual mean time series were calculated across 3 trials for each outcome variable. Data were analyzed for trials where the participant landed with a subsequent maximal jump: that is, upward-pointing black arrow for DVJ and upward-pointing black arrow as well as downward-pointing red arrow for DVJcog.

In addition to landing mechanics, individual mean jump height was calculated as the displacement of the center of mass of the pelvis from standing to peak height during the jump across the 3 trials using the Visual 3D software. Jump height was included to complement the landing mechanics outcomes with a measure of lower limb explosive power.

### Statistical Analyses

The time series data were statistically analyzed using functional independent *t* tests based on the interval-wise testing procedure within the functional data analysis framework, which is a nonparametric, permutation-based method.^29^ Instead of analyzing individual data points, these methods statistically analyze the complete time series as a single unit and, in our case, identify the specific areas where differences between the groups are observed. To ensure more robust findings, we used interval-wise, testing-adjusted *P* values to assess statistical significance within each analysis. Results were considered significant only if they covered domains representing at least 5% of the profile; any significant findings in smaller domains were not interpreted.^25^

Comparisons were made between the injured and uninjured limb for the ACLR group to the matched dominant and nondominant legs (defined as the preferred leg to kick a ball as far as possible) for the uninjured controls. Jump height was analyzed between the groups using a univariate linear model with sex included as a covariate to account for differences in jump height between male and female participants. All statistical analyses were conducted using R (Version 3.6.1), with a 5% alpha level set a priori.

## Results

### Landing Mechanics

#### DVJ

Individuals with ACLR displayed significantly (adjusted *P* < .05) less hip power (62-74 ms; 1.14-1.20 W/kg less) for their injured leg compared with controls. Individuals with ACLR also displayed more hip abduction moment (30-69 ms; 0.11-0.17 N·m/kg more) for the uninjured leg compared with controls. No further between-group differences were observed for the remaining outcomes (Appendix Figure A1, available in the online version of this article). Comparisons of individual mean curves within the ACL group according to time from injury to ACLR and time from ACLR to testing revealed no time-dependent effects on the DVJ time series.

#### DVJcog

For upward-pointing black arrows, individuals with ACLR displayed, for the injured leg, significantly (adjusted *P* < .05) less hip power (82-96 ms; 1.28-1.39 W/kg less), knee power (83-92 ms; 3.21-3.29 W/kg less), knee flexion moment (84-95 ms; 0.21-0.24 N·m/kg less), ankle power (17-41 ms; 1.78-2.47 W/kg less), and ankle dorsiflexion moment (19-85 ms; 0.08-0.20 N·m/kg less) compared with controls. Similar to their DVJ performance, individuals with ACLR also displayed more hip abduction moment (31-71 ms; 0.13-0.20 N·m/kg more) for the uninjured leg compared with controls. The remaining outcomes revealed no significant between-group differences (Appendix Figure A2, available online).

For downward-pointing red arrows (also indicating a subsequent jump), individuals with ACLR displayed, for the injured leg, significantly (adjusted *P* < .05) less knee power (81-95 ms; 3.10-3.40 W/kg less), knee flexion moment (85-98 ms; 0.21-0.25 N·m/kg less), ankle power (14-44 ms; 1.69-2.61 W/kg less), and ankle dorsiflexion moment (27-63 ms; 0.13-0.19 N·m/kg less) compared with controls. Similar to their DVJ performance, individuals with ACLR also displayed more hip abduction moment (39-68 ms; 0.15-0.20 N·m/kg more) for the uninjured leg compared with controls. The remaining outcomes revealed no significant between-group differences (Appendix Figure A3, available online). Comparisons of individual mean curves within the ACL group according to time from injury to ACLR and time from ACLR to testing revealed no time-dependent effects on the DVJcog time series.

### Jump Height

For DVJ, the jump height was not statistically significant between groups (*P* = .083). However, a greater and significant between-group difference in jump height was observed for DVJcog, both for black arrows (ACLR mean 0.033 m lower; *P* = .026) and for red arrows (ACLR mean 0.041 m lower; *P* = .006) (Table 2).

**Table 2:** Jump Height in Meters for the Different Arrow Conditions During the Drop Vertical Jumps*^*a*^*

	ACLR	Controls	ACLR-Controls	*P* ^*b*^
DVJ black arrow up	0.401 ± 0.093	0.425 ± 0.094	–0.025 (0.003 to −0.053)	.083
DVJcog black arrow up	0.377 ± 0.095	0.411 ± 0.088	–0.033 (–0.004 to −0.063)	.026
DVJcog red arrow down	0.369 ± 0.096	0.410 ± 0.088	–0.041 (–0.012 to −0.070)	.006

a ACLR, anterior cruciate ligament reconstruction; DVJ, drop vertical jump; DVJcog, drop vertical jump with secondary cognitive tasks. Data are expressed as mean ± SD or mean (95% CI).

b Sex was included as a covariate in analyses to account for differences in jump height.

## Discussion

We investigated differences in landing mechanics and jump height between individuals with ACLR compared with uninjured controls for DVJs with and without secondary cognitive tasks. Our hypothesis that adding secondary cognitive tasks to the DVJs would show greater between-group differences in lower limb landing mechanics and jump height compared with the standard DVJ was confirmed for kinetics but not for kinematics. Specifically, the ACLR group showed, for the injured leg, less knee power, knee flexion moment, ankle power, and ankle dorsiflexion moment and also lower jump height than controls for the DVJ with cognitive dual tasks (DVJcog) but not for the standard DVJ. The ACLR group also showed significantly less hip power for the injured leg and more hip abduction moment for the uninjured leg compared with controls, with these differences remaining fairly consistent regardless of whether secondary cognitive tasks were added to the DVJ.

The between-group differences during DVJcog, characterized by reduced loading of the injured leg and decreased jump height compared with controls, are considered an indication of incomplete rehabilitation.^21^ The significantly lower hip, knee, and ankle power, along with reduced knee flexion and ankle dorsiflexion moments, suggest force generation and absorption deficits. Impaired control and absorption of ground-reaction forces may elevate the risk of reinjury or injury to the other ACL and hinder sports performance, particularly in scenarios requiring fast decision making and motor execution. The persistent compensatory increase in hip abduction moment for the uninjured leg further highlights asymmetric loading, which could predispose individuals to secondary musculoskeletal issues over time. Although kinematic analyses revealed no significant group differences, trends suggest asymmetric landing mechanics in individuals with ACLR. A more neutral frontal plane hip and knee alignment on the injured leg indicated a protective strategy, whereas the noninjured leg showed higher hip abduction moments and a tendency for increased knee adduction moments. These compensatory patterns may affect long-term joint health and rehabilitation outcomes.

The significantly lower jump height observed in DVJcog also suggests that cognitive demands exacerbate performance deficits in individuals with ACLR, indicating enhanced CMi. This may impair their ability to perform in real-world sporting situations, where attention must be divided between movement execution and environmental cues. In contrast, the smaller between-group difference in jump height for the standard DVJ approaching statistical significance (*P* = .083) suggests that such impairments may be less evident during isolated jump tasks. This underscores the value of incorporating dual-task assessments in rehabilitation and RTS screening.

Some recent studies have compared the influence of neurocognitive challenges on unplanned landing mechanics among individuals with and without ACL injury. Alanazi et al^2^ observed no significant differences between groups (ACLR group 5.0 ± 3.3 years after surgery) in peak hip, knee, and ankle flexion angles or (external) flexion moments when a secondary task of heading a football was added during the flight phase of a forward jump. The lack of differences may have been due to the simplicity of the secondary task, which lacked cognitive complexity.^2^ In contrast, Gholipour Aghdam et al^13^ observed greater changes in landing mechanics (eg, greater vGRF and less knee flexion) for individuals with ACLR at a mean 42.8 ± 10.0 months after surgery compared with controls when participants attempted to align their landing foot with 1 of 3 colored circles depending on the color illuminated in front of them when leaving the box they began standing on. Gokeler et al^14^ recently found that maximal single-leg hop distance decreased significantly yet similarly for individuals with ACLR at a mean 11.1 ± 4.5 months after surgery and controls when adding a secondary cognitive task challenging working memory. Despite the lack of between-group differences for hop distance, kinematics from inertial measurement units revealed significant increases in the variability of joint coordination during landing for both legs of controls and the uninjured but not the injured leg of the ACLR group. Gokeler and colleagues suggested that the lack of changes seen in the injured leg was due to an overreliance on cognitive control when attempting to maintain stability during landing. This lack of adaptability was further suggested to increase secondary ACL injury risk due to a lack of flexibility in movement coordination, which is required in chaotic sporting environments.^14^

Thus, despite methodological differences between ours and the aforementioned studies (eg, cognitive task complexity, movement analysis methods, and bilateral vs unilateral motor tasks), the combined findings indicate that individuals with ACL injury show greater CMi than controls. Greater CMi among athletes with a history of ACLR compared with uninjured athletes has also been revealed through poorer cognitive and motor performances during dual-task DVJ testing.^31^ Greater CMi may increase the risk for secondary ACL injury during sport due to the necessity to maintain knee control while adapting to the chaotic sporting environment.^15,17^ Further research on CMi as a consequence of ACL injury is warranted, preferably targeting both movement mechanics and performance outcomes for the motor and cognitive tasks. However, a key question is whether deficits in landing mechanics during dual-task testing increase primary injury risk or whether an ACL injury drives such impairment. Research shows that athletes who later sustained an ACL injury performed worse on traditional neuropsychological tests than those who remained injury-free.^33^ However, cognitive-motor function in dual-task settings appears largely independent of traditional neuropsychological and motor tests, as shown in noninjured athletes.^37^ This underscores the need for further research with a prospective design including testing among uninjured cohorts with long-term follow-up and repeated testing during ACL rehabilitation.

Previous research involving DVJs without secondary cognitive tasks also revealed less loading of the injured limb for individuals with a history of ACLR. Meyer et al^26^ reported that individuals with ACLR at a mean 8.9 ± 1.3 months after surgery demonstrated less knee sagittal plane energy absorption and less (although nonsignificant) peak knee flexion moment for their injured leg compared with controls. Similar findings were revealed in a study by Mueske et al,^27^ where individuals with ACLR at a mean 6.5 ± 1.8 months after surgery exhibited lesser flexion moments and energy absorption at the knee and ankle although greater flexion moments and energy absorption at the hip as well as greater knee abduction moments.

Our study involved participants with a longer time after ACLR, a mean 24.9 ± 16.1 months, revealing similar between-group findings that reached statistical significance only when secondary cognitive tasks were introduced. This may be attributed to the partial normalization of landing mechanics over time after ACLR, as demonstrated by significant improvement in DVJ landing mechanics symmetry between 8 and 25 months after ACLR in young athletes.^18^ Our results therefore suggest that the lesser loading of the ACLR side reported by Meyer et al^26^ and Mueske et al^27^ would have been enhanced with added secondary cognitive tasks. Furthermore, the significantly lower jump height for our ACLR group compared with our controls was observed only when secondary cognitive tasks were added, thus indicating that decrements in motor performance due to neurocognitive challenges may be exacerbated by the injury. However, these results stand in contrast to those of Gokeler et al,^14^ who found that decreases in single-leg hop distance when adding reaction and working memory tasks were not significantly different between individuals with a history of ACLR and uninjured controls. Further research, ideally using single-leg tasks with secondary cognitive tasks, is needed to establish between-group differences in hop performances.

Clinically, our findings of additional biomechanical differences between groups for the DVJcog that were not observed for the standard DVJ support recommendations for including neurocognitive challenges in rehabilitation and RTS testing.^7,15-17^ We argue that DVJcog better reflects the demands of attending and adapting to the environment found in both recreational and elite sports compared with the standard DVJ, thus demonstrating better ecological validity.^5,15^ Our results of more pronounced between-group differences during DVJcog also indicate a better sensitivity in assessing movement mechanics as part of RTS screening. A recent systematic review found that CMi can be mitigated among uninjured athletes across a range of motor tasks after cognitive-motor dual-task training.^38^ These findings highlight the value of identifying individuals with aberrant motor control under dual-task conditions so that appropriate interventions can be implemented to improve performance and, it is hoped, reduce injury risk. However, intervention studies are required among individuals with ACL injury to ascertain whether such training can lead to clinically meaningful adaptations. Designing such training is not straightforward, given the complexity in combining a high-impact motor task with complex secondary cognitive tasks that achieve an appropriate difficulty level and desired motor learning.

### Strengths and Limitations

This study has several strengths and limitations worth noting. One strength is the use of functional data analysis methods with interval-wise testing-adjusted *P* values, which provided robust and significant results within specific domains during the first 100 ms of landing. This phase is particularly relevant given that noncontact ACL injuries are estimated to occur within approximately 15 to 50 ms after impact.^6,20,23^

Our participants performed a bilateral task. A single-leg task would more closely resemble noncontact ACL injury mechanisms and may be more likely to reveal aberrant landing mechanics. Future research should thus investigate the influence of cognitive tasks on single-leg mechanics to facilitate comparisons between the injured and uninjured legs. Also, the cognitive tasks of the current study targeted fast decision making, inhibitory control, attention, and short-term memory. These aspects were included to mimic challenges that are experienced in sporting environments. It remains unclear, however, whether the use of arrows results in similar behavior compared with the cognitive demands of sport, which is more complex considering factors such as the athlete-environment relationship with associated constraints.^5^ Future research should focus on advancing this area through continued dual-task test development and the design of interventions that improve CMi and movement mechanics, aiming to clarify their role as critical factors in ACL rehabilitation.

We did not apply strict inclusion criteria, resulting in varied participant characteristics. The participants performed diverse sports (although rapid directional changes characterized all), had different times between ACLR and testing, and followed different rehabilitation programs under the guidance of external clinicians. Although all participants received a hamstring graft (in line with national practice), we had no access to medical records and could screen for concomitant injuries only by interviewing the participants before testing. Our results therefore apply to the general population for whom these factors are relevant.

## Conclusion

Individuals with ACLR demonstrated significant differences in multiple outcomes of landing mechanics as well as lower jump height compared with uninjured controls when secondary cognitive tasks were added to DVJs. Importantly, most of these differences were not observed for a standard DVJ without secondary cognitive tasks. The aberrant landing mechanics indicate an incomplete rehabilitation. Our findings thus suggest a need to provide neurocognitive challenges in rehabilitation and screening for RTS to improve rehabilitation outcomes and reduce reinjury rates.

## Supplemental Material

sj-pdf-1-ajs-10.1177_03635465251346145 – Supplemental material for Neurocognitive Challenges During Drop Vertical Jumps Increase Sensitivity to Differentiate Atypical Landing Mechanics and Jump Height in Individuals With Anterior Cruciate Ligament Reconstruction Supplemental material, sj-pdf-1-ajs-10.1177_03635465251346145 for Neurocognitive Challenges During Drop Vertical Jumps Increase Sensitivity to Differentiate Atypical Landing Mechanics and Jump Height in Individuals With Anterior Cruciate Ligament Reconstruction by Andrew Strong and Jonas L. Markström in The American Journal of Sports Medicine
