# Supplementary material for: Neurocognitive Challenges During Drop Vertical Jumps Increase Sensitivity to Differentiate Atypical Landing Mechanics and Jump Height in Individuals With Anterior Cruciate Ligament Reconstruction
Source: Am J Sports Med. 2025 Jun 7;53(9):2154–61. doi: 10.1177/03635465251346145 (PMC12235058; doi:10.1177/03635465251346145)
Supplement: sj-pdf-1-ajs-10.1177_03635465251346145 – Supplemental material for Neurocognitive Challenges During Drop Vertical Jumps Increase Sensitivity to Differentiate Atypical Landing Mechanics and Jump Height in Individuals With Anterior Cruciate Ligament Reconstruction [file sj-pdf-1-ajs-10.1177_03635465251346145.pdf]

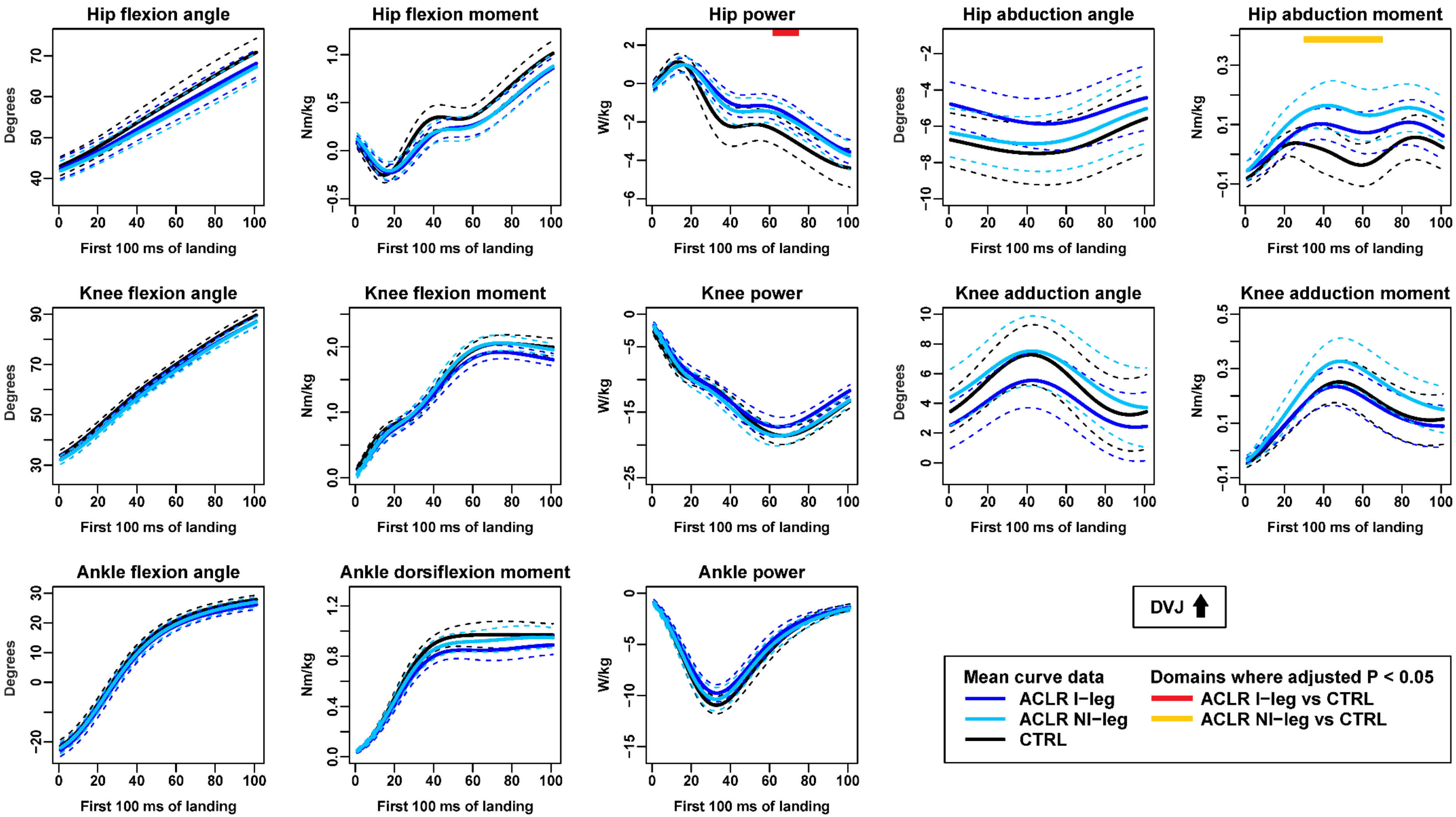

Appendix Figure 1. Mean hip, knee, and ankle kinematic and kinetic time series with 95% confidence intervals during the first 100 ms of DVJ landing (black arrows) with horizontal bars indicating domains with significant differences between groups.

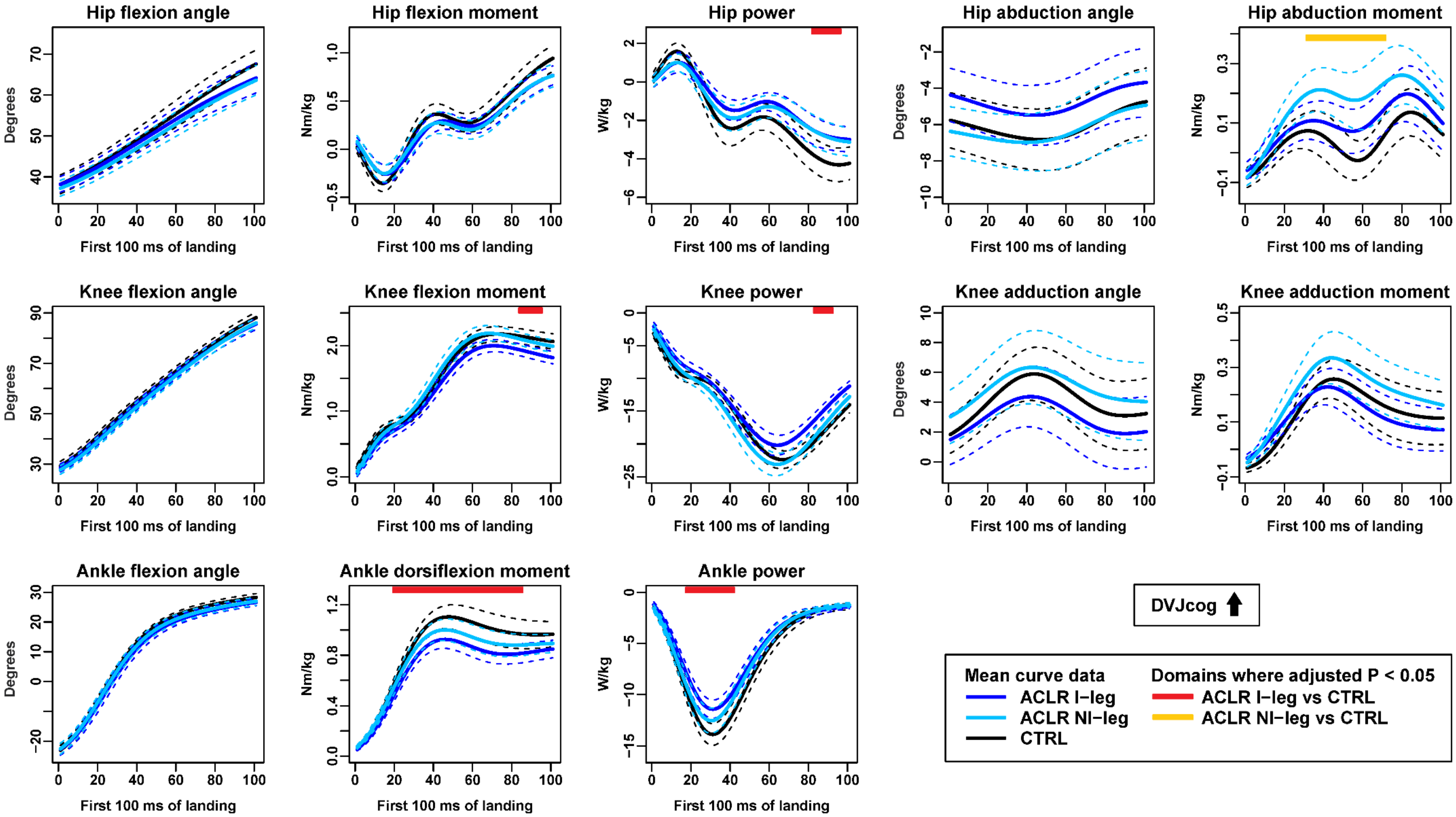

Appendix Figure 2. Mean hip, knee, and ankle kinematic and kinetic time series with 95% confidence intervals during the first 100 ms of DVJcog landing (black arrows) with horizontal bars indicating domains with significant differences between groups.

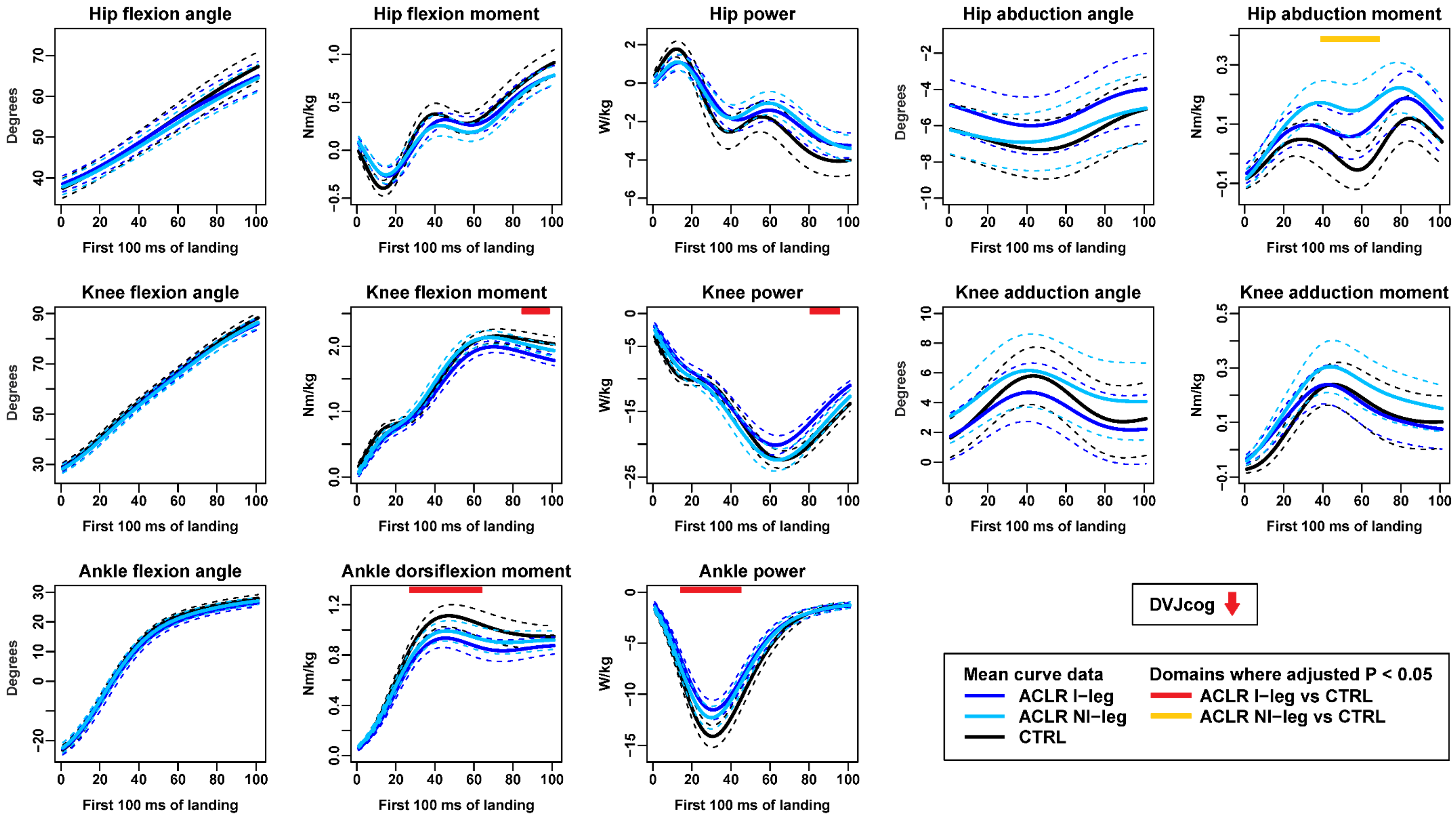

Appendix Figure 3. Mean hip, knee, and ankle kinematic and kinetic time series with 95% confidence intervals during the first 100 ms of DVJcog landing (red arrows) with horizontal bars indicating domains with significant differences between groups
